# A heteroskedastic error covariance matrix estimator using a first-order conditional autoregressive Markov simulation for deriving asympotical efficient estimates from ecological sampled *Anopheles arabiensis *aquatic habitat covariates

**DOI:** 10.1186/1475-2875-8-216

**Published:** 2009-09-21

**Authors:** Benjamin G Jacob, Daniel A Griffith, Ephantus J Muturi, Erick X Caamano, John I Githure, Robert J Novak

**Affiliations:** 1 School of Medicine, University of Alabama, Birmingham, Birmingham AL, USA; 2 School of Social Sciences, University of Texas, Dallas, TX, USA; 3 Human Health Division, International Centre of Insect Physiology and Ecology (ICIPE), Nairobi, Kenya

## Abstract

**Background:**

Autoregressive regression coefficients for *Anopheles arabiensis *aquatic habitat models are usually assessed using global error techniques and are reported as error covariance matrices. A global statistic, however, will summarize error estimates from multiple habitat locations. This makes it difficult to identify where there are clusters of *An. arabiensis *aquatic habitats of acceptable prediction. It is therefore useful to conduct some form of spatial error analysis to detect clusters of *An. arabiensis *aquatic habitats based on uncertainty residuals from individual sampled habitats. In this research, a method of error estimation for spatial simulation models was demonstrated using autocorrelation indices and eigenfunction spatial filters to distinguish among the effects of parameter uncertainty on a stochastic simulation of ecological sampled *Anopheles *aquatic habitat covariates. A test for diagnostic checking error residuals in an *An. arabiensis *aquatic habitat model may enable intervention efforts targeting productive habitats clusters, based on larval/pupal productivity, by using the asymptotic distribution of parameter estimates from a residual autocovariance matrix. The models considered in this research extends a normal regression analysis previously considered in the literature.

**Methods:**

Field and remote-sampled data were collected during July 2006 to December 2007 in Karima rice-village complex in Mwea, Kenya. SAS 9.1.4^® ^was used to explore univariate statistics, correlations, distributions, and to generate global autocorrelation statistics from the ecological sampled datasets. A local autocorrelation index was also generated using spatial covariance parameters (i.e., Moran's Indices) in a SAS/GIS^® ^database. The Moran's statistic was decomposed into orthogonal and uncorrelated synthetic map pattern components using a Poisson model with a gamma-distributed mean (i.e. negative binomial regression). The eigenfunction values from the spatial configuration matrices were then used to define expectations for prior distributions using a Markov chain Monte Carlo (MCMC) algorithm. A set of posterior means were defined in WinBUGS 1.4.3^®^. After the model had converged, samples from the conditional distributions were used to summarize the posterior distribution of the parameters. Thereafter, a spatial residual trend analyses was used to evaluate variance uncertainty propagation in the model using an autocovariance error matrix.

**Results:**

By specifying coefficient estimates in a Bayesian framework, the covariate number of tillers was found to be a significant predictor, positively associated with *An. arabiensis *aquatic habitats. The spatial filter models accounted for approximately 19% redundant locational information in the ecological sampled *An. arabiensis *aquatic habitat data. In the residual error estimation model there was significant positive autocorrelation (i.e., clustering of habitats in geographic space) based on log-transformed larval/pupal data and the sampled covariate depth of habitat.

**Conclusion:**

An autocorrelation error covariance matrix and a spatial filter analyses can prioritize mosquito control strategies by providing a computationally attractive and feasible description of variance uncertainty estimates for correctly identifying clusters of prolific *An. arabiensis *aquatic habitats based on larval/pupal productivity.

## Background

The autoregressive conditional variance (i.e., nuisance parameter) is important in mapping *Anopheles arabiensis *Patton, as it is used in habitat prediction and confidence intervals, tests of hypotheses, spectral estimates, and for estimating prediction error in the model [1]. Nuisance parameters are often variances, but there are exceptions: for example, in an errors-in-variables model, generated from *An. arabiensis *aquatic habitat parameter estimates, the unknown true habitat location of each observation is a nuisance parameter [2]. Stochastic models have been generated with non-linear nuisance parameters for examining the interrelationship between mosquito productivity and oviposition of gravid mosquitoes [3]. By designing a model that explicitly features non-stationary behavior of *An. arabiensis *aquatic habitat data, a hierarchy of conditional variance components can be linked by applying Bayes theorem [4-6]. Commonly, having obtained the joint conditional distribution of all of the unknown random variables, given the known sampled habitat covariates, by applying Bayes theorem, nuisance variables are marginalized to obtain the conditional distribution for determining ecological parameters associated with georeferenced anopheline aquatic habitat data. However, even though this generalized treatment of the conditional variance can generate an autoregressive error model, the residual estimates will not be able to spatially target prolific *An. arabiensis *aquatic habitats based on larval/pupal productivity. Treatments of anopheline aquatic habitat perturbations should be based on surveillance of larvae in the most productive areas of an ecosystem [1,2]. Additionally, residual-based diagnostics for multivariate heteroscedasticity from previously constructed *An. arabiensis *aquatic habitat models has revealed that errors in variance uncertainty estimation can substantially alter numerical predictions of a model by inflating the value of test statistic thereby, increasing the chance of a Type I error - incorrect rejection of the null hypothesis, *H*_0_*: no spatial autocorrelation *[1,2]. Autocorrelation is a characteristic of data derived from a process that is articulated in one or more spatial dimensions which can describe the error structure of ecological sampled data [2]. Thus, autoregression forecasts of *An. arabiensis *aquatic habitat locations requires an absolute relative prediction error estimator to identify prolific habitats for developing habitat-based intervention models for implementing Integrated Vector Management (IVM).

Traditionally, the random error terms in Gaussian autoregressive models have been posited as a proper conditional autoregressive (PCAR) or as an improper conditional autoregressive (ICAR) specification for identifying spatial trends in residual parameter estimates [10]. The normal distribution in these models furnishes a feasible prior distribution for coefficients while the error variance prior distribution often is represented in the gamma distribution. Statistical criteria in autoregressive coefficients are crucially dependent on such assumptions as normality and homogeneity [11]. However, the CAR prior is usually improper, making it imperative to constantly check the propriety of the joint posterior [12]. Even though this problem can be remedied by introducing a constrained autoregressive parameter to ensure a proper joint distribution for a resulting multivariate model, input errors and structural data errors still can give rise to complex error structures, including heteroscedasticity and nonstationarity. Maximum likelihood estimation which ignores heteroskedasticity yields inconsistent estimates of the variance-covariance matrix and renders likelihood ratio tests with restrictions which make assumptions of the Gauss-Markov theorem of independence among sampled habitat covariates inappropriate [13]. These prediction errors can lead to overconfidence in the estimates of parameter values, or to errors in an *An. arabiensis *aquatic habitat model being compensated by large residual variances.

For determining spatial errors in an *An. arabiensis *aquatic habitat model, Bayesian geostatistical kriging models of the form described in Diggle et al. [5] has on occasion been used as opposed to the CAR model. The Bayesian kriging model assumes that autoregressive errors are modeled using a multivariate Gaussian distribution with an uncertainty covariance matrix expressed as a parametric function of the distance between pairs of georeferenced data points. Another uncertainty estimator, for spatial simulation models generated from field and remote-sampled *An. arabiensis *aquatic habitat parameters, is a relative error norm technique which normalizes the difference between model predictions and sampled predictor variables and computes residual estimates for discrete and continuous domain problems [14]. Using this technique, multiplicative errors can be treated in the same way as in an autoregressive model using log-transformed habitat data (i.e., larval/pupal counts). The error model is then evaluated using predictions based on some optimal parameter set. Another metric involves measuring uncertainty estimates through statistical distributions and classical hypothesis testing [15]. Examples of the metric includes the Bayesian Model Averaging (BMA) [16], which for ecological sampled *An. arabiensis *aquatic habitat covariates can be applied by directly likelihood weighting the outputs of multivariate analyses either by using deterministic or stochastic techniques. Subsequently, the predictive error distributions obtained with these models can be combined using BMA techniques to obtain a multi-model prediction of *An. arabiensis *aquatic habitat locations.

Although relative norm and BMA can explicitly model the covariance structure of the error terms in an *An. arabiensis *aquatic habitat model, the output will be in the form of global parameter estimates. These global estimates can indicate how reliable results from an *An. arabiensis *aquatic habitat model are but, like any global statistic these accuracy assessments will summarize the standard error from many sampled habitat locations. Standard heuristic approach to anopheline aquatic habitat model selection is to measure when global residual error variance begins to stabilize [7]. However, global statistics will summarize standard error from many sampled habitat locations, thus making it difficult for spatial assessment of predictive error at a single sampled habitat. Moreover, if global parameter estimates are used for evaluating autoregressive residual coefficients, then the assumption is that parasitological indicators of *An. arabiensis *aquatic habitats are homogenous in their quantitative predictions. For example, the assumption must be made that contacts between hosts and blood feeding mosquitoes are uniformly distributed in the focal area, whereas studies has shown blood feedings of mosquitoes tend to aggregate in geographic space [1,2].

Local spatial autocorrelation indices [17,18] may provide a method for assessing variance uncertainty estimates in models generated from field and remote-sampled of *An. arabiensis *aquatic habitats covariates. By far, the most popular test for spatial autocorrelation is based on the Moran *I *test statistic. In essence, this test statistic is formulated as a properly normalized quadratic in terms of the variables that are being tested for spatial correlation. Moran's original specification standardizes the variables by subtracting the sample mean, and then deflating by an appropriate factor. The error variance-covariance matrix appearing in the quadratic form, based on the non-independence of the sampled observations, is a spatially weighted matrix. The eigendecomposition of this matrix may have interesting properties in various contexts for mapping variance uncertainty in Bayesian probabilistic models using distribution properties of Moran's *I *and generalized linear models. Algorithms that assume independently-distributed errors of *An. arabiensis *aquatic habitats may formally establish an asymptotic distribution of the Moran test statistic for determining spatial correlation in models for quantifying variance uncertainty estimates.

In this research, error propagation in Bayesian regression coefficients was spatially quantified using Monte Carlo Markov Chain (MCMC) methods, and ecological parameters of individual sampled riceland *An. arabiensis *aquatic habitats. The MCMC methods are a class of powerful stochastic algorithms, which provides a means for taking spatially dependentsamples from probability distributions, by generating a set of random samples from an arbitrary probability density function (pdf), which in Bayesian analysis is the posterior distribution [8]. Essentially all inference about uncertainty in Bayesian regression models, generated from ecological sampled covariates of anopheline aquatic habitats have revealed high reliability in their prediction estimates [3]. Spatial filtering techniques were then used, which included the eigendecomposition of a spatial weighted matrix, using the non-linear regression estimates generated from the Bayesian framework. Spatial eigendecomposition models can focus on an error specification, at the habitat level, using a mean response that forces the auto-model spatial dependency parameter value to zero [1,2]. In this research, the eigenvector filtering approach promoted by Griffith et al [17] and Getis and Griffith [18] was used, which is a non-parametric technique that removes the inherent spatial autocorrelation from generalized linear regression models by treating it as a missing variables (i.e., first order) effect. The aim of non-parametric spatial filtering is to control for residual latent autocorrelation at the individual habitat level, with a set of proxy variables rather than to identify a global autocorrelation parameter for a spatial process [19]. An autoregressive variance uncertainty analyses for heteroskedastic error modeling was then performed using autocorrelation indices in which conditional means and residual variances were specified. Given valid assumptions about the nature of variance uncertainty estimates in Bayesian applications, autocorrelating error residuals in a spatial weights matrix may provide a method for predicting clusters of *An. arabiensis *aquatic habitats.

Additionally, testing variance uncertainty estimates from a spatial autocorrelation error matrix may reveal pertinent statistics (e.g., y-intercept, slope coefficients, standard errors, t-values, residuals, and diagnostic test results) for determining the relative plausibility of a model for correctly statistically prioritizing sampled covariates of *An. arabiensis *aquatic habitats based on larval/pupal productivity. These statistical approaches may also infer correlates of species abundance data (Poisson or normally distributed response), for other mosquito species and insect research, while accounting for spatial autocorrelation in model error residuals using autocovariate regression, spatial eigenvector mapping, generalized least squares (conditional and simultaneous) autoregressive models and generalized estimating equations. Therefore, our objectives in this research were to: (1) generate global autocorrelation statistics for decomposing sampled *An. arabiensis *aquatic habitat parameters into spatial eigenvectors using a Poisson model with a gamma-distributed mean; (2) perform a Bayesian regression analyses incorporating a MCMC algorithm using field and remote sampled predictor variables; and, (3) autocorrelate all uncertainty coefficients in a spatially weighted matrix to determine variance uncertainty in an *An. arabiensis *aquatic habitat model.

## Methods

### Field sampling strategy

**Figure 1:**
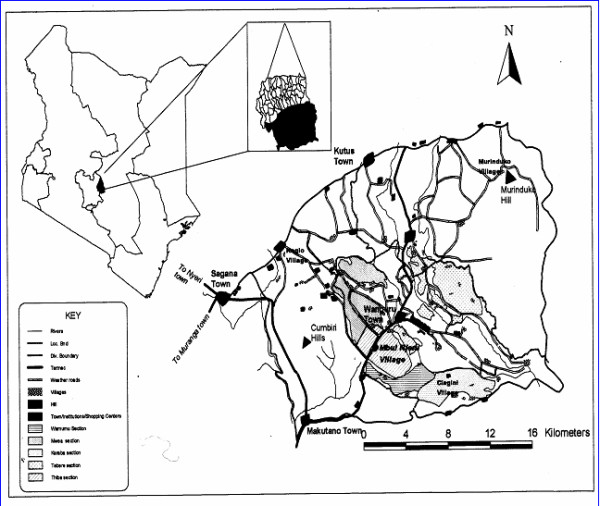
**Base map of the Karima study site**.

The sampling strategy used for the collection of immature *An. arabiensis *aquatic habitat data was developed for earlier research projects and has been described in detail elsewhere [[1,20,21], and [22]]. Base maps were prepared for the study site in ArcGIS (Figure 1). We expected the larval/pupal count in *An. arabiensis *aquatic habitats in the study site to follow a Poisson distribution, as was the case in previous research in other Kenyan areas [[1,2], and [3]]. Therefore, the mean count and standard deviations was used, on the log-number of mosquito larval/pupal counts collected in the study site, to determine sample size requirements. A sampling intensity formula was applied for determining the number of *An. arabiensis *aquatic habitats to collect when randomly sampling from an infinite population *n *= (ts/E)^^2^, where t = t value (t = 2), s = the standard deviation of log-larval/pupal count values observed, (s = 0.889), and E was the desired half-width of the confidence interval around the mean expressed in same units as standard deviation (E = ln(1.25) [1,2]. Applying this formula, it was determined that 152 samples were required. The vector image of the sampling scheme (grid cell) was overlaid with the land cover raster images to identify areas of interest within each polygon (grid cell) of the sampling scheme. All potential aquatic habitat sites were identified, and data relative to species composition and abundance, predators, water quality and other environmental variables were assessed.

### Poisson Regression

A Poisson regression, with statistical significance, was determined by a 95% confidence level which was used to ascertain whether the proportions of riceland aquatic habitats, positive for *An. arabiensis *larvae/pupae, differed by sampled grid cell. Poisson regression can be used for prediction (including forecasting of time-series data), inference, hypothesis testing, and modeling of causal relationships among riceland *An. arabiensis *aquatic habitat covariates [1,2,20-22]. The regression analyses assumed independent counts (i.e., *n*_*i*_), taken at habitat locations *i *= 1 2... *n*, where each of the sampled *An. arabiensis *aquatic larval/pupal count values, was from a Poisson distribution. These larval/pupal counts were described by a set of explanatory variables denoted by matrix **X**_*i*_, a 1×*p *vector of covariate estimates for a sampled habitat location *i*. The expected value of these data was given by:

(2.3)

where β was the vector of non-redundant parameters and the Poisson rates parameter was given by:

(12.4)

The rates parameter *λ*_*i *_(**X**_*i*_) was both the mean and the variance of the Poisson distribution for an *An. arabiensis *aquatic habitat *i*. The dependent variable was the total larval/pupal count in an *An. arabiensis *aquatic habitat. The regression analyses were performed in SAS PROCREG. The sampled habitat data were log-transformed before analyses to normalize the distribution and minimize standard error. All of the covariate estimates for the models were tested for multicollinearity, using partial F test in SAS, and no problematic correlations were found.

### Bayesian estimation procedures

In this research Bayesian estimation and MCMC methods were used to model the sampled covariates of *An*. *arabiensis *aquatic habitats in the study site. In the Bayesian paradigm, hierarchical models can be used to model heterogeneity of variances on the log- scale [8]. In this research, the natural logarithms of variances were modeled using a linear model to account for heterogeneity of the variances (on a logarithmic scale), in terms of the predictor variables sampled. In an anopheline aquatic habitat model, an environment-specific variance parameter is considered to be an independent draw from a random sampling distribution [3].

The MCMC sampling began with conditional (marginal) probability distributions, and the parameter estimates that were obtained using pseudo-likelihood estimation (i.e., an autoregressive term estimated with a conventional regression procedure). This involved estimating covariate coefficients (β) and *ρ *as though the field and remote-sampled observations were independent. MCMC outputs can sample values for an anopheline aquatic habitat parameter drawn from the joint posterior probability distribution [3]. In the first stage of the Bayesian analyses, a likelihood model was specified for the vector of the *An. arabiensis *aquatic habitats larval/pupal counts. At the second stage, predictor variables of the sampled *An. arabiensis *aquatic habitats were analyzed for specifying a prior model.

WinBUGS^® ^was used to recognize conjugate specifications (e.g. Poisson-gamma), from the field and remote-sampled mosquito data. Our model assumed that the number of larval/pupal counts in the study site, *i, Y*_*i*_, had a conditional independent Poisson distribution with mean *E*_*i *_exp (*μ*_*i*_). The variable *E*_*i *_was used as the expected number of sampling events, which was proportional to the corresponding known *An. arabiensis *aquatic habitat larval/pupal population, *n*_*i*_. The expression exp (*μ*_*i*_) was the relative risk based on the sampled larval/pupal count values: regions with exp (*μ*_*i*_) > 1 having greater numbers of observed *An. arabiensis *aquatic habitat larval/pupal count values than expected, and vice versa for regions with exp (*μ*_*i*_) < 1, at the study site. The log-relative term was *μ*_*i *_which modeled all the predictor variables of *An. arabiensis *aquatic habitat data, linearly as:

(2.1)

In this research, **x'**_*i *_was the sampled *An. arabiensis *aquatic habitat covariates, and β was a vector of fixed effects in the models. Additionally, the terms *θ*_*i *_and *φ*_*i *_were used for capturing site-specific random effects and spatial dependence, respectively, in the ecologically sampled datasets. In previous research, Jacob et al [3] employed an MCMC algorithm and an autocovariate matrix to spatially quantify stochastic error propagation in Bayesian parametric variables estimated from *Anopheles gambiae *s.l. aquatic habitat covariates sampled in Malinda and Kisumu, Kenya. Their models revealed that a 10 cm increase in habitat depth was associated with a 0.391 cm increase in larval/pupal count on average, but after adjusting for habitat depth in both urban sites, using the spatial regression models, no significant autocorrelation or clustering of *An. gambiae *s.l. aquatic habitats appeared present in the residual error estimates. In this research all site specific *An. arabiensis *aquatic habitat characteristics were imposed using the equations:

(2.2)

Three chains were estimated for the variables in each potential model. Samples were discarded to allow the model to stabilize and the next 10,000 samples, after burn in, were used to derive parameter estimates. Discarding the first set of "burn-in" iterations can ensure that the chain has reached steady state, when estimating Monte Carlo parameters, such as posterior means from sampled anopheline habitat covariates [3]. After the model had converged, samples from the conditional distributions were used to summarize the posterior distribution of the model.

The Monte Carlo method of error propagation assumed that the distribution of error variables for each of the input data layers, generated in WinBUGS^® ^from the ecological sampled *An. arabiensis *aquatic habitats parameters, were known. For each of the data layers an error surface was simulated by drawing, at random, from an error pool defined by the geographic distribution of the sampled habitat data. Error surfaces were added to the input data layers and the model was run using the resulting data error layers as input. The process was repeated so that, for each run, a new realization of an error surface was generated for each input data layer. The results of each run were accumulated and a running mean and standard deviation surface for the output was calculated. This process continued until the running mean stabilized. Since the random error visualizations were both positive and negative, the stable running mean were taken as the true model output surface, and the standard deviation surface was used as a measure of relative error. A simple summary was generated, showing posterior mean, median and standard deviation, with a 95% posterior credible interval.

Models were compared using the Deviance Information Criterion (DIC) in WinBUGS^®^, where , was the sum of the posterior mean of the deviance, *(D)*, a measure of goodness-of-fit, and the effective number of parameters (*p*_*D*_), a measure of model complexity. A measure of goodness-of-fit based on the DIC values was applied and an *R*^2^DIC, calculated in line with the standard *R*^2 ^measure for the regression models. This was defined as:  where DIC_*k *_was the DIC value for model *k *under evaluation, DIC_max _was the DIC value for one-fixed parameter model and  was the posterior deviance from the model [3].

### Checking the statistical efficiency of the MCMC Sequence

Model checking of all data input and compilation was also conducted in WinBUGS^®^. The number of chains had to be specified before compilation. In this research, three parallel chains were run. Syntax checking was used, which involved highlighting the entire model code and then choosing the sequence model specification. The uncertainty in estimates of quantities derived from an MCMC sequence of random samples was represented by *N*_*k *_and habitat samples *v*_*k *_represented a pdf of a scalar quantity *v*. The estimated value of *v *was given by the sample mean,



In this research, the expected variance in  was the expectation for the ensemble of the sequences generated from the ecological sampled *An. arabiensis *aquatic habitats covariates which was expressed as:



where . The autocovariance of the sequence was defined as: . The normalized autocovariance was , where *σ*^2 ^was the variance of *v *and *ρ *(*l*) did not depend on *k*. The length of the nonzero normalized autocovariance values were:



The normalized autocovariance was a symmetric function, i.e. *ρ *(-*l*) = *ρ *(*l*). The sequence sufficiently converged to the target pdf. The variance of the distribution of the sampled habitat parameters was generated using:



and the normalized autocovariance was estimated from the sequence using:



for lag *l *≥ 0.

The MCMC sequence was defined as the reciprocal of the ratio of the number of MCMC trials needed to achieve the same variance in an estimated quantity as are required for independent draws from the target probability distribution [3]. The estimation of the mean and the variance for independent sampled *An. arabiensis *aquatic habitat parameters were generated by:



After compilation, the files contained some initial values for the parameters selected in the model. After careful inspection of the data, no aberrant values, leading to numerical overflow were found.

### Spatial autocorrelation error matrix

All residual estimates from the Bayesian model were then evaluated in a spatial error (SE) model. An autoregressive model was employed that used a sampled habitat variable, Y, as a function of nearby sampled habitat Y values [i.e., an autoregressive response (AR) or spatial linear (SL) specification] and/or the residuals of Y as a function of nearby Y residuals [i.e., an AR or SE specification]. Distance between sampled habitats was defined in terms of an *n*-by-*n *geographic weights matrix, **C**, whose *c*_*ij *_values were 1 if the sampled *An. arabiensis *aquatic habitat locations *i *and *j *were deemed nearby, and 0 otherwise. Adjusting this matrix by dividing each row entry by its row sum, with the row sums given by **C1**, converted this matrix to matrix **W **[19].

The *n*-by-1 vector *x *= [*x*_1 _⋯ *x*_*n*_]^*T *^contained measurements of a quantitative variable for *n *spatial units and *n*-by-*n *spatial weighting matrix **W**. The formulation for the Moran's index of spatial autocorrelation used in this research was:



where  with *i *≠ *j*

The values *w*_*ij *_were spatial weights stored in the symmetrical matrix **W **[i.e., (*w*_*ij *_= *w*_*ji*_)] that had a null diagonal (*w*_*ii *_= 0). In this research the matrix was initially generalized to an asymmetrical matrix **W**. Matrix **W **can be generalized by a non-symmetric matrix W* by using *W *= (W* + W*^T^)/2 [19]. Moran's *I *was rewritten using matrix notation:



where H = (*I *- 11^*T*^/*n*) was an orthogonal projector verifying that *H *= *H*^2^, (i.e., *H *was independent). Features of matrix **W **for analyzing sampled covariates of *An. arabiensis *aquatic habitats include that it: is a stochastic matrix, expresses each observed value y_i _as a function of the average of habitat location *i*'s nearby habitat larval/pupal counts, and allows a single spatial autoregressive parameter, *ρ*, to have a maximum value of 1 [1].

### Simultaneous autoregressive model (SAR) specifications

A SAR model specification was used to describe the autoregressive variance uncertainty estimates. A spatial filter (SF) model specification was also used to describe both Gaussian and Poisson random variables. The resulting SAR model specification took on the following form:

(2.1a)

where *μ *was the scalar conditional mean of Y, and ε was an *n*-by-1 error vector whose elements were statistically independent and identically distributed (iid) normally random variates. The spatial covariance matrix for equation (2.1), using the sampled anopheline aquatic habitat covariates was E [(**Y **- *μ***l**)' (**Y **- *μ***l**)] = Σ = [(**I **- *ρ ***W**')(**I **- *ρ ***W**)]^-1^σ^2^, where E (●) denoted the calculus of expectations, **I **was the *n*-by-*n *identity matrix denoting the matrix transpose operation, and *σ*^2 ^was the error variance.

However, when a mixture of positive and negative spatial autocorrelation is present in an *An. arabiensis *aquatic habitat model, a more explicit representation of both effects leads to a more accurate interpretation of empirical results [1]. Alternately, the excluded values may be set to zero, although if this is done then the mean and variance must be adjusted [19]. In this research, two different spatial autoregressive parameters appeared in the spatial covariance matrix *An. arabiensis *aquatic habitat model specification, which for an SAR model specification became:

(2.2a)

where the diagonal matrix of autoregressive parameters, <ρ >_diag_, contained two sampled parameters: *ρ*_+ _for those *An. arabiensis*. aquatic habitat pairs displaying positive spatial dependency, and *ρ*. for those habitat pairs displaying negative spatial dependency. For example, by letting *σ*^2 ^= 1 and employing a 2-by-2 regular square tessellation,



for the vector ,

enabled positing a positive relationship between the sampled *An. arabiensis *aquatic habitats by covariates, y_1 _and y_2_, a negative relationship between covariates, y_3 _and y_4_, and, no relationship between covariates y_1 _and y_3 _and between y_2 _and y_4_. This covariance specification yielded:

(2.3a)

where **I**_+ _was a binary 0-1 indicator variable which denoted those *An. arabiensis *aquatic habitat covariates displaying positive spatial dependency, and **I**_- _was a binary 0-1 indicator variable denoting those sampled habitats displaying negative spatial dependency, using **I**_+ _+ **I**_- _= **1**. Expressing the preceding 2-by-2 example in terms of equation (2.3) yielded:



If either *ρ*_+ _= 0 (and hence **I**_+ _= **0 **and **I**_- _= **I**) or *ρ*_- _= 0 (and hence **I**_- _= **0 **and **I**_+ _= **I**), then equation (2.3) reduces to equation (2.1) [19]. This indicator variables classification was made in accordance with the quadrants of the corresponding Moran scatterplot generated using the sampled *An. arabiensis*. aquatic habitat covariates sampled in the study site.

### The SF model specification

If positive and negative spatial autocorrelation processes counterbalance each other in a mixture, the sum of the two spatial autocorrelation parameters--(ρ_+ _+ *ρ*.) will be close to 0 [19]. In this research, Jacobian estimation was implemented by utilizing the differenced indicator *An. arabiensis *aquatic habitat variables (**I**_+ _- γ **I**_-_), estimating *ρ*_+ _and *γ *with maximum likelihood techniques, and setting . The Jacobian generalizes the gradient of a scalar valued function of multiple variables which itself generalizes the derivative of a scalar-valued function of a scalar [17]. A more complex *An. arabiensis*. aquatic habitat specification was then posited by generalizing these binary indicator variables. We used *F*: *R*^*n *^→ *R*^*m *^as a function from Euclidean *n*-space to Euclidean *m*-space which was generated using the distance between sampled *An. arabiensis *aquatic habitat covariates. Such a function was given by *m *habitat covariate (i.e., component functions), *y*_1_(*x*_1_, *xn*), *y*_*m*_(*x*_1_, *xn*). The partial derivatives of all these functions were organized in an *m*-by-*n *matrix, the Jacobian matrix *J *of *F*, which was as follows:



This matrix was denoted by *J*_*F *_(*x*_1_,..., *x*_*n*_) and . The *i *th row (*i *= 1,..., *m*) of this matrix was the gradient of the *i*^*th *^component function *y*_*i*_:(∇ *y*_*i*_). In this analyses **p **was a sampled *An. arabiensis *aquatic habitat covariate in *R*^*n *^and *F *(i.e., sampled larval/pupal count) was differentiable at **p**; its derivative was given by *J*_*F*_(*p*). The model described by *J*_*F*_(*p*)) was the best linear approximation of *F *near the point **p**, in the sense that:

(2.4)

The spatial structuring was achieved by constructing a linear combination of a subset of the eigenvectors of a modified geographic weights matrix, using (**I **- **11**'/n) **C **(**I **- **11**'/n) that appeared in the numerator of the Moran's Coefficient (MC) Spatial autocorrelation can be indexed with a MC, a product moment correlation coefficient [19]. A subset of eigenvectors was then selected with a stepwise regression procedure. Because (**I **- **11**'/n) **C **(**I **- **11**'/n) = **E Λ E'**, where **E **is an *n*-by-*n *matrix of eigenvectors and Λ is an *n*-by-*n *diagonal matrix of the corresponding eigenvalues [17], the resulting *An. arabiensis *aquatic habitat model specification was given by:

(2.5)

where *μ *the scalar mean of Y, Ek was an *n*-by-k matrix containing the subset of k <<*n *eigenvectors selected with a stepwise regression technique, and β was a *k*-by-1 vector of regression coefficients [18]

A number of the eigenvectors were extracted from (**I **- **11**'/n) **C **(**I **- **11**'/n), which were affiliated with geographic patterns of the sampled *An. arabiensis *aquatic habitat covariates, in the study site, portraying a negligible degree of spatial autocorrelation. Consequently, only k of the *n *eigenvectors was of interest for generating a candidate set for a stepwise regression procedure. Candidate eigenvector represents a level of spatial autocorrelation which can account for the redundant information in orthogonal anopheline aquatic habitat map patterns [1]

The preceding eigenvector properties resulted in  and  for equation (2.3). Expressing equation (2.3) in terms of the preceding 2-by-2 example yielded



Of note is that because the 2-by-2 square tessellation rendered a repeated eigenvalue.

### Surface partitioning

To identify spatial clusters of *An. arabiensis *aquatic habitats, Thiessen polygon surface partitioning were generated to construct geographic neighbor matrices, which also were used in the spatial autocorrelation analysis. Entries in matrix were 1, if two sampled *An. arabiensis *aquatic habitats shared a common Thiessen polygon boundary and 0, otherwise. Next, the linkage structure for each surface was edited to remove unlikely geographic neighbors to identify pairs of sampled *An. arabiensis *aquatic habitats sharing a common Thiessen polygon boundary. Attention was restricted to those map patterns associated with at least a minimum level of spatial autocorrelation, which, for implementation purposes, was defined by |MC_j_/MC_max_| > 0.25, where MC_j _denoted the *j*th value and MC_max_, the maximum value of MC. This threshold value allowed two candidate sets of eigenvectors to be considered for substantial positive and substantial negative spatial autocorrelation respectively. These statistics indicated that the detected negative spatial autocorrelation may be considered to be statistically significant, based upon a randomization perspective. Of note, is that the ratio of the PRESS (i.e., predicted error sum of squares) statistic to the sum of squared errors from the MC scatterplot trend line was 1.27 which was well within two standard deviations of the average standard prediction error value (roughly 1.13) for a sampled *An. arabiensis *aquatic habitat in the study site. Because larval/pupal counts were being analyzed, a Poisson spatial filter model specification was employed in this research [1,2]. Detected overdispersion (i.e., extra-Poisson variation) results in its mean being specified as gamma distributed [19].

The model specification was written as follows:



where *μ*_*i *_was the expected mean larval/pupal count for habitat location *i*, μ was an *n*-by-1 vector of expected larval/pupal counts, LN denoted the natural logarithm (i.e., the generalized linear model link function), *α *was an intercept term, and *η *was the negative binomial dispersion parameter. This log-linear equation had no error term; rather, estimation was executed assuming a negative binomial random variable.

### Eigenfunctions of a spatial weighting matrix

The upper and lower bounds for a spatial matrix generated using Morans indices (*I*) can be given by *λ*_max_(n/1^*T*^*W*1) and *λ*_min_(n/1^*T*^*W*1) where *λ*_max _and *λ*_min _which are the extreme eigenvalues of Ω = *HWH *[23]. Hence, in this research, the eigenvectors of Ω were vectors with unit norm maximizing Moran's *I*. The eigenvalues of this matrix were equal to Moran's *I *coefficients of spatial autocorrelation post-multiplied by a constant. Eigenvectors associated with high positive (or negative) eigenvalues have high positive (or negative) autocorrelation [19]. The eigenvectors associated with eigenvalues with extremely small absolute values correspond to low spatial autocorrelation and are not suitable for defining spatial structures [17]

The diagonalization of the spatial weighting matrix generated from the field and remote-sampled *An. arabiensis *aquatic habitat covariate coefficients consisted of finding the normalized vectors *u*_*i*_, stored as columns in the matrix *U *= [*u*_1 _⋯ *u*_*n*_], satisfying:



where Λ = *diag *(*λ*_1 _⋯ *λ *_*n*_),  and  for *i *≠ *j*. Note that double centering of Ω implied that the eigenvectors *u*_*i *_generated from the ecological sampled *An. arabiensis *aquatic habitat covariates were centered and at least one eigenvalue was equal to zero. Introducing these eigenvectors in the original formulation of Moran's index lead to:

(2.6)

Considering the centered vector *z *= *Hx *and using the properties of idempotence of *H*, equation (2.6) was equivalent to:

(2.7)

### From autocorrelation to correlation coefficient

As the eigenvectors *u*_*i *_and the vector *z *were centered, equation (2.7) was rewritten:

(2.8)

In this research, *r *was the number of null eigenvalues of Ω (*r *≥ 1). These eigenvalues and corresponding eigenvectors were removed from Λ and *U *respectively. Equation 2.8 was then strictly equivalent to:

(2.9)

Moreover, it was demonstrated that Moran's index for a given eigenvector *u*_*i *_was equal to *I*(*u*_*i*_) = (*n*/1^*T *^*W*1)*λ *_*i *_so the equation was rewritten:



The term *cor*^2 ^(*u*_*i*_, *z*) represented the part of the variance of *z *that was explained by *u*_*i *_in the *An. arabiensis *aquatic habitat model *z *= *β *_*i *_*u*_*i*_*+ e*_*i*_. This quantity was equal to . By definition, the eigenvectors *u*_*i *_were orthogonal, and therefore, regression coefficients of the linear models *z *= *β *_*i *_*u*_*i*_*+ e*_*i *_were those of the multiple regression model *z *= *Uβ *+ *ε *= *β *_*i*_*u*_*i *_+ ⋯ + *β *_*n*-*r *_*u*_*n*-*r *_+ *ε*.

### The distribution of the error residuals in the autocovariance matrix

The maximum value of *I *was obtained by all of the variation of *z*, as explained by the eigenvector *u*_1_, which corresponded to the highest eigenvalue *λ*_1 _in the spatial autocorrelation error matrix. In this research, *cor*^2 ^(*u*_*i*_, *z*) = 1 (and *cor*^2 ^(*u*_*i*_, *z*) = 0 for *i *≠ 1) and the maximum value of *I*, was deduced for Equation (2.9), which was equal to *I*_max _= *λ*_1_(*n*/1^*T*^*W*1). The minimum value of *I *in the error matrix was obtained as all the variation of *z *was explained by the eigenvector *u*_*n*-*r *_corresponding to the lowest eigenvalue *λ*_*n-r *_generated in the *An. arabiensis *aquatic habitat model. This minimum value was equal to *I*_min _= *λ*_*n*-*r *_(*n*/1^*T*^*W*1). If the ecological sampled predictor variable was not spatialized, the part of the variance explained by each eigenvector was equal, on average, to *cor*^2 ^(*u*_*i*_, *z*) = 1/*n*-1. Because the field and remote-sampled *An. arabiensis *aquatic habitat variables in *z *were randomly permuted, it was assumed that we would obtain this result. In this research the set of *n*! random permutations, revealed that . It was easily demonstrated that  and it followed that .

## Results

Table 1 lists the dependent and independent variables collected in the study site. Table 2 lists the improvements of fit in the adjusted and unadjusted models. The most parsimonious model was selected as the "final" model. The information in Table 3 indicated that aquatic animals count, canopy cover over habitat, and rice stage status all significantly improved model fit. Table 4 presents the results of the Poisson regression performed in SAS^® ^for the interactions model. These results provided information for estimates of the prior distribution of main effect coefficients for the Bayesian analysis performed in WinBUGS^®^.

**Table 1:** Information collected in the rice fields of Karima study site for analysis in SAS

**Variable**	**Description**	**Units**
An count	Total larval count (dependent variable)	Count

Tillers	Density	Number/Square meter

Depth	Field depth	Centimeters

Canopy	Canopy cover	Percent

Turbidity	Turbidity status	0 = not turbid, 1 = turbid

Disanimal	Distance to animal	Meters

**Table 2:** Comparison of improvement of fit measured by likelihood ratio between unadjusted and adjusted effects models, and full main effects and interactions and saturated models for the Karima study site

**Unadjusted effects**				**Adjusted effects**		
**Variable**	**Deviance**	**Improvement *χ*^2^**	**df**	**Deviance**	**Improvement *χ*^2^**	**df**

Intercept	996.9673					

DANIMAL	981.9554	15.0119	1	901.4757	20.0341	1
TILLERS	983.6985	13.2688	1	885.147	3.7054	1

CANOPY	988.6662	8.3011	1	890.101	8.6594	1

TURBIDITY	987.6537	11.5043	1	891.752	9.3862	1

DEPTH	986.8716	10.0957	1	901.9639	20.5223	1

1^st ^Degree Interactions				844.8677	38.9132	5

**Table 3:** Improvement of Fit of the WinBUGS Hierarchical Bayesian Model (HBM) model

**Unadjusted effects**			**Adjusted efferts**	
**Variable**	**df**	**Improvement *χ*^2^**	**Improvement *χ*^2^**	**df**

DANIMAL	1	-1.368	-0.353	1
TILLERS	1	6.089	3.242	1

CANOPY	1	1.187	1.432	1

**Table 4:** Results of SAS regression used to estimate prior distribution of coefficients for WinBUGS MCMC analysis

**Variable**	**df**	**Coefficient**	**SE**	**P**
Intercept	1	1.4020	0.1053	<0.0001

DANIMAL	1	0.0357	0.0057	<0.0001

TILLERS	1	0.0052	0.0066	0.4297
CANOPY	1	0.0172	0.0044	<0.0001

TURBIDITY	1	0.0483	0.0341	<0.0001

DEPTH	1	0.0521	0.1702	0.7596

The values for parameter estimates and standard errors in Table 5 were used as mean values and standard errors to parameterized prior expected values for the habitat covariates. The prior expected mean value for the error term was assumed to be zero ('0') with a standard deviation of 0.01. Initial values for the MCMC chains were automatically generated by WinBUGS^®^. The first 1,000 samples were discarded to allow the model to stabilize and the next 10,000 samples were used to derive parameter estimates. Median parameter values as well as the 95% credibility intervals (2.5 percentile and 97.5 percentile values).

**Table 5:** Coefficient parameters estimates for WinBUGS Bayesian model

**Variable**	**Mean**	**SD**	**MC error**	**2.5%**	**50%**	**97.5%**
Intercept	1.427	0.0804	0.0013	1.267	1.427	1.581

TILLERS	0.018	0.0091	0.0001	0.001	0.017	0.033

**Figure 2:**
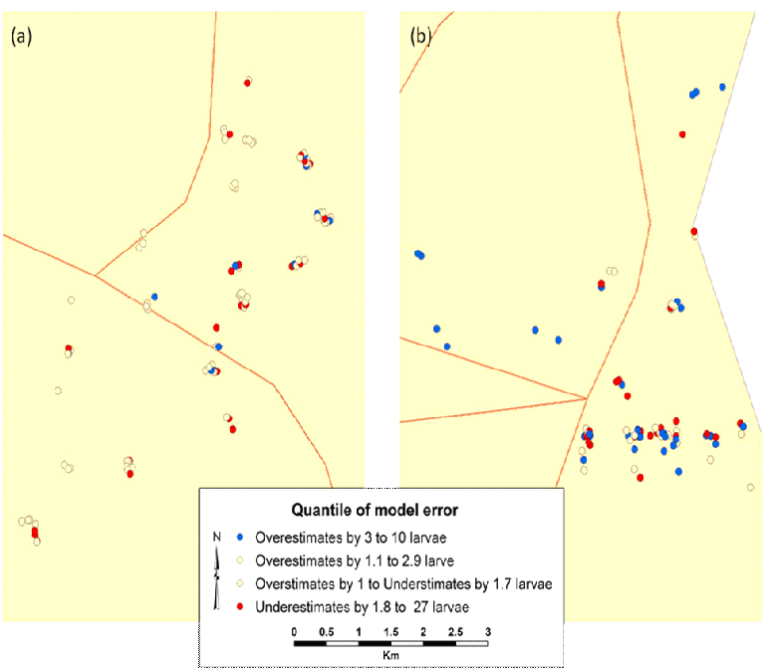
**Spatially adjusted error estimates for ecological sampled *An. arabiensis *aquatic habitats the Karima study site**.

The DIC value for the model was 924.3. The DIC values indicated that the final model, containing number of tillers and study site fit better than the alternative model with only study site. Smaller DIC value indicates a better model [8]. Similarly, the final model fit better than the full main effects model containing aquatic animal count, number of tillers, canopy cover over habitat, rice stage and study site which had a DIC value of 927.2. As a sampled *An. arabiensis *aquatic habitat increased in number of tillers in the study site the median log-count of larvae/pupae increased 0.031. The spatially adjusted model that assumed independence among the field and remote predictor variables of *An. arabiensis *root mean square error (RMSE) fit better that the spatially non-unadjusted model than the for correlation within a study site (Figure 2).

Table 6 presents the spatial analysis of residual errors and number of tillers for the study site. After adjusting for number of tillers using regression outputs, significant clustering of *An. arabiensis *aquatic habitats appeared present in the residual error estimates. The distribution of the residual error appeared non-random. The spatial autocorrelation error covariance matrix identified the sampled covariate depth of habitat as a significant predictor of *An. arabiensis *aquatic habitat larval/pupal count values.

**Table 6:** Spatial analysis of residual errors and habitat depths for the Karima study site

**Statistic**	**Study site**
Raw Count data (unadjusted for habitat factors)	Karima

Moran's I Coefficient (Z)	0.654 (0.341)
Residual Error	

Moran's I Coefficient (Z)	-0.058 (-1.060)

Depth of habitat	

Moran's Coefficient I (Z)	0.048 (1.342)

Estimation results from SAS PROC GENMOD for the model appear in Table 7. Positive and negative spatial autocorrelation spatial filter component pseudo-R^2 ^values are reported. These values do not exactly sum for the complete spatial filter; however, the values are very close to their corresponding totals, suggesting that any induced multicollinearity was quite small. The spatial autocorrelation components suggested the presence of approximately 19% redundant information in the *An. arabiensis *larval/pupal count samples.

**Table 7:** Poisson spatial filtering model results for *Anopheles arabiensis *larval mosquito counts by study site

**Spatial statistics**	**Karima**
SF: # of eigenvectors	8

SF: MC	0.03

SF: GR	0.71
SFpseudo-R^2^	0.30

Positive SA SF: # of eigenvectors	1
Positive SASF: MC	.922

Positive SA SF: GR	0.08

Positive SA SF pseudo-R^2^	0.08

Negative SA SF: # of eigenvectors	7

Negative SA SF: MC	-0.52

Negative SA SF: GR	0.60

Negative SA SF pseudo-R^2^	0.22

Deviance statistic	1.08

Dispersion parameter	0.16

MC: Moran's Coefficient

GR: Geary's Ratio

SF: spatial filter

SA: spatial autocorrelation

## Discussion

In the Bayesian analyses, all "high risk" habitats were identified and ranked based on the sampled ecological covariates and larval/pupal productivity. Parameter estimates were used to define expectations for prior distributions in the autoregressive framework, which revealed that the sampled covariate number of tillers was a significant predictor variable, positively associated with *An. arabiensis *aquatic habitats in the study site. The abundance of *An. arabiensis *has been associated with early vegetative stage of the rice growth [20,21]. At the tillering stage, in Karima rice fields, there is addition of inorganic nitrogenous fertilizers [24]. The addition of the nitrogenous fertilizers can act as the attractant for oviposition by gravid *An. arabiensis *mosquitoes. Broadcasting nitrogenous fertilizers in rice fields has been found to enhance mosquito larval populations [24,25]. For effective control of developmental stages of mosquito larvae, the application of larvicides should be done at the vegetative stage and the larvicides should persist until the beginning of the reproductive stage of the rice [21].

The summarization of the simulated posterior distribution correctly accounted for the error of estimation of all sampled *An. arabiensis *aquatic habitat parameters; each simulated posterior distribution represented an "average" over the joint posterior distributions of all other parameters in the model so that any uncertainty estimation of the sampled predictor variables was fully accounted for in both the mean or the mode of simulated posteriors and in the dispersion of the posterior. Because Bayesian statistical analysis is involved, prior distributions need to be posited for each varying quantity: the response variable, each variable coefficient, the spatial autoregressive parameter, the error variance, and the random error term [8]. This "Bayesian averaging" over the uncertainty of estimation is a very desirable property of Bayesian frameworks for modeling *An. arabiensis *aquatic habitat covariates as any predictor variable, depending strongly on poorly estimated parameters, will have relatively flat posteriors i.e., the posterior will be a direct indication that the available precision on the parameter is very poor. In this research, the DIC comprised two goodness-of-fit measures and the posterior distribution of the deviance, which was the number of effective parameters for measuring complexity in the *An. arabiensis *aquatic habitat model. Aquatic habitats with high larval/pupal count, were compared with the results of a Monte Carlo simulation, which established the probabilities and occurrences of highly productive habitats in the study site based on larval/pupal productivity.

The spatial filter analyses used geographic weights matrices and a stepwise negative binomial regression routine, to select eigenvectors as regressors. This eigenvector spatial filtering approach added a minimally sufficient set of eigenvectors as proxy-variables to a set of linear predictors of *An. arabiensis *aquatic habitats. The regression residuals represented spatially independent variable components. The eigenvectors yielded distinct *An. arabiensis *aquatic habitat map patterns for description of the latent autocorrelation in the sampled data. There was positive autocorrelation in the residual spatial pattern: similar log-larval/pupal counts of *An. arabiensis *aquatic habitats aggregated in geographic space based on the sampled covariate depth of habitat.

Positive autocorrelation pattern in *An. arabiensis *aquatic habitat covariates is often driven by multiple causes that may be exogenous (e.g. autocorrelated environment disturbance) and/or endogenous (conspecific attraction, dispersal limitations, demography) [1,2]. For example, positive autocorrelation patterns of anopheline aquatic habitats can be influenced by environmental landscape [26], vector control activities [27], host density [28], proximity to larval habitats and blood-meal hosts [20], quality of the larval habitats [21], availability of domestic animals [22] and inter-human variation in mosquito preferences, based on host odors and other cues [24]. Positive autocorrelation may be also due to common local weather patterns that cause habitats to spatially cluster and partially govern anopheline larval/pupal population dynamics [1,2]. Climatic factors particularly temperature, precipitation and relative humidity, predicts to a large degree the natural distribution of *An. arabiensis *aquatic habitats [1], as well as ecological factors, such as predation, parasitism, cannibalism, availability of blood meal hosts and quality of larval habitats [2]. Additionally, mosquito species differ in their habitat preference and disproportionately utilize available aquatic habitats. For example, some species, such as *An. funestus*, thrive in permanent and marshy water bodies [20] and others, including *An. gambiae *and *An. arabiensis*, prefer small pools of water that are sun-lit and devoid of vegetation [28]. Mosquitoes also differ in their foraging behavior, as well as host choice and resting behavior [21], which can effect clustering of *An. arabiensis *aquatic habitats based on larval/pupal productivity. Furthermore, socio-economic/demographic dimensions in riceland environments may tend to impact upon contagion diffusion, inducing *An. arabiensis *aquatic habitats to cluster together in geographic space [1]. For example, the number of sleepers, the house roof materials (grass thatch, iron, or tile roof) have significant effects on the number of mosquitoes caught [2].

A graduated, systematic MCMC sampling methodology that uses a spatial autocorrelation error matrix for Gaussian variance estimation, can adjust for sampled ecological covariates, which can identify more clustering of *An. arabiensis *aquatic habitats within riceland areas than techniques that use a random sampling strategy. A major advantage of using autocorrelation indices is that the sampling error distributions are well-defined. Thus, if the epidemiological data about the hotspots (clusters of *An. arabiensis*) is correct, based on targeted MCMC surveillance, then using autocorrelation indices for variance uncertainty estimation can yield model outputs with higher sensitivity for detection of highly productive *An. arabiensis *aquatic habitats, than random surveillance for riceland larval control operations. The statistical significance of spatio-temporal autocorrelation patterns found in the model can be directly assessed using standard normal deviates (*z *scores). Since it is more feasible to expand intensified surveys to targeted *An. arabiensis *aquatic habitats, based on spatially selected potential foci [1], a systematic MCMC surveillance sampling frame, using a spatial autocorrelation error matrix for estimating variance uncertainty, can focus on specific habitats, which would allow for intensified entomologic surveillance at prolific habitats, while not increasing overall sampling efforts. Random interventions are excessive and wasteful because the vectors are not themselves randomly distributed [3].

A spatial autocorrelation error matrix can also locally calculate total, omission and commission errors using one assessment and report each conditional-variance term in the model using the original sampled data units. These error residuals will be reported as one value for each sampled habitat location rather than being a combination of habitat values. The ability to adequately reflect the spatial dependence in individual sampled habitat covariates comprehensively in an important advantage of using autocorrelation indices for variance uncertainty estimation in an *An. arabiensis *aquatic habitat model. The strategy of targeted interventions is to recognize the importance of the variation in mosquito production among individual habitat breeding sites throughout the rice cycle [1]. Autocorrelation indices should not be interpreted, however, as a direct estimate of the correlation parameter: a spatial stochastic model, such as a first-order conditional autoregressive (spatial Markov) model, must first be specified to enable parameter estimation. Although in this research the discussion was centered on malaria vectors, specifically of the *An. gambiae *complex, the framework and derived guidelines described are applicable to integrated control programs for other mosquito species and insect born diseases.

## Conclusion

The Bayesian regression analyses revealed that the sampled covariate number of tillers was positively associated with prolific *An. arabiensis *aquatic habitats based on larval/pupal productivity in the study site. A spatial filter analyses selected eigenvectors as regressors, resulting in spatial autocorrelation being filtered out of the residuals of the ecological sampled data. The spatial filtering analyses transformed all variables, containing spatial dependence, into covariates free of spatial dependence by partitioning the original georeferenced *An. arabiensis *aquatic habitat attribute variable, within a generalized linear model framework, into two synthetic variates: (1) a spatial filter variate capturing latent spatial dependency, that otherwise would have remained in the response residuals, and (2) a nonspatial variate that was free of spatial dependence. The eigenfunction spatial filter derived from the MC determined the mean, variance and statistical distribution characterizations and descriptions of the sampled covariates at each individual habitat. The spatial autocorrelation residual error analyses using the estimates from the Monte Carlo simulation suggested positive autocorrelation of the *An. arabiensis *aquatic habitats based on the covariate depth of habitat. The spatial autocorrelation error matrix revealed the presence of roughly 19% redundant information in the *An. arabiensis *aquatic habitat parameter estimates. The spatially adjusted models identified the clustering patterns of the sampled *An. arabiensis *aquatic habitat in the ecological datasets while accounting for all conditional heteroscedastic error terms in the models. Autocorrelation indices can enable significance testing of *An. arabiensis *aquatic habitat models using field and remote sampled explanatory variables which can be very useful for model improvement and resource allocation for implementing mosquito control strategies in riceland areas.

## Competing interests

The authors declare that they have no competing interests.

## Authors' contributions

BGJ conceived the study and led the drafting of this manuscript; DG, EJ and EC contributed to the interpretation and results of the statistical models. JG supervised the field data collection and helped analyze the data; RJ is the principal investigator of the study. All authors interpreted the results and wrote the paper.
